# Ancient genomics

**DOI:** 10.1098/rstb.2013.0387

**Published:** 2015-01-19

**Authors:** Clio Der Sarkissian, Morten E. Allentoft, María C. Ávila-Arcos, Ross Barnett, Paula F. Campos, Enrico Cappellini, Luca Ermini, Ruth Fernández, Rute da Fonseca, Aurélien Ginolhac, Anders J. Hansen, Hákon Jónsson, Thorfinn Korneliussen, Ashot Margaryan, Michael D. Martin, J. Víctor Moreno-Mayar, Maanasa Raghavan, Morten Rasmussen, Marcela Sandoval Velasco, Hannes Schroeder, Mikkel Schubert, Andaine Seguin-Orlando, Nathan Wales, M. Thomas P. Gilbert, Eske Willerslev, Ludovic Orlando

**Affiliations:** Centre for GeoGenetics, Natural History Museum of Denmark, University of Copenhagen, Copenhagen, Denmark

**Keywords:** ancient DNA, genomics, next generation sequencing

## Abstract

The past decade has witnessed a revolution in ancient DNA (aDNA) research. Although the field's focus was previously limited to mitochondrial DNA and a few nuclear markers, whole genome sequences from the deep past can now be retrieved. This breakthrough is tightly connected to the massive sequence throughput of next generation sequencing platforms and the ability to target short and degraded DNA molecules. Many ancient specimens previously unsuitable for DNA analyses because of extensive degradation can now successfully be used as source materials. Additionally, the analytical power obtained by increasing the number of sequence reads to billions effectively means that contamination issues that have haunted aDNA research for decades, particularly in human studies, can now be efficiently and confidently quantified. At present, whole genomes have been sequenced from ancient anatomically modern humans, archaic hominins, ancient pathogens and megafaunal species. Those have revealed important functional and phenotypic information, as well as unexpected adaptation, migration and admixture patterns. As such, the field of aDNA has entered the new era of genomics and has provided valuable information when testing specific hypotheses related to the past.

## 1. The impossible genome

Ancient DNA (aDNA) research is full of surprises. Less than a decade ago, most experienced aDNA researchers believed that full genome sequencing of extinct species such as the woolly mammoth and Neandertals was impossible. The best available technology at the time was incredibly demanding in terms of fossil material, experimental work load and cost. First, each piece of target genomic DNA had to be amplified several times by PCR, then ideally PCR amplicons had to be propagated using bacterial vectors, and a number of clones had to be sequenced before a consensus sequence devoid of sequencing errors could be generated [1]. Furthermore, this whole procedure often needed to be replicated in another laboratory, before DNA sequences could be considered authentic [2].

The size of PCR amplifiable fragments was most often limited to approximately 100–150 base pairs (bp) at best, which represented little sequence information. With exceptionally well-preserved samples, the characterization of the whole approximately 16.5 kilobases (kb) of mitochondrial genomes [3–5] could be achieved using overlapping amplicons [6], but nuclear markers [7,8] were more difficult to amplify owing to their lower copy number per cell. Considering usual aDNA concentrations, each microlitre of DNA extract yielded one PCR amplicon at best. Therefore, it was generally necessary to destructively sample large amounts of fossil material to sequence complete mitochondrial genomes. The sequencing of the cave bear mitochondrial genome required for instance 1 g of bone material and not less than 570 PCR amplicons [5]. Assuming approximately 3 gigabases as the size of the nuclear genome and similar PCR success rates for mitochondrial and nuclear templates, this technology would have required approximately 180 kg of material and more than 103 million amplicons to generate a first draft of the cave bear genome. Given standard PCR and sequencing times, even on platforms with the highest throughput at the time (384 reactions per run), this would have required approximately 48 000 years of experimental work, excluding the time required for cloning! The characterization of even the best-preserved woolly mammoth specimens would have required similar efforts [4]. Two-round multiplex reactions [9], whereby a set of PCR targets are co-amplified from the same microlitre of DNA extract, could help reduce material and time requirements by one or two orders of magnitude [10,11], but operational costs would still amount to billions of US dollars. In summary, this technology limited palaeogenomics to the shorter ancient microbial genomes [12].

## 2. Shotgun sequencing of the first ancient mammalian genome

An alternative approach consisted of shotgun sequencing following aDNA ligation into bacterial plasmids [13,14]. This could be done at large sequencing centres, though with two major limitations. First, most of the sequences generated in fact do not originate from the organism of interest, but from environmental microbes that colonize the tissue after deposition. Therefore, no more than 26.9 kb of the cave bear genome could be reconstructed with this approach from a total of 14 027 sequences [13]. The second limitation was the heavy experimental load required for bacterial cloning. The invention of ‘emulsion PCR’, whereby each DNA library template is amplified in a water–oil emulsion droplet, and the development of the 454 platform provided a time-effective alternative to bacterial cloning by processing hundreds of thousands of sequencing reactions in parallel [15]. Applied to DNA extracts from an approximately 28 000 years (28 kyr) old mammoth bone sample, this technology provided, in a 6 h long run, 28 megabases (Mb) of metagenomic data, of which approximately 13 Mb belonged to the mammoth genome [16]. This demonstrated for the first time that sequencing of complete mammalian genomes was probably achievable from realistic amounts of bone material.

The field improved further, with the realization that hair constitutes a remarkable source of high-quality aDNA [17,18] that could be subjected to efficient decontamination procedures [19] inapplicable to bones. Deep sequencing of DNA from ancient mammoth hair yielded approximately 80% of sequences identified as being of mammoth origin [20], thus providing a first draft covering approximately 70% of the mammoth genome, with an overall sequencing error rate estimated at 0.345%. These data revealed that 99.4% of the sequenced mammoth genome was identical to the African elephant genome and identified 29 mammoth genes with specific non-synonymous mutations of potential functional importance. Testing this list of gene candidates following the methodology that revealed the association between an allelic variant at the MC1R gene and blond coat-colour [9] could illuminate our understanding of the genetic make-up of mammoths.

## 3. The first ancient human genome

By the time the first draft of the mammoth genome was characterized, new sequencing technologies with higher throughput were available [21]. The Illumina Genome Analyzer II platforms could generate 180 million sequence reads per run. This massive sequencing throughput, combined with the high endogenous DNA content of hair, and preservation in a cold environment made the sequencing of the first ancient human genome possible. The individual sequenced was a palaeo-Eskimo belonging to the Saqqaq culture, who lived along the southwestern coast of Greenland 4 kyr ago [22]. The sequence information gathered represented an average depth of 20-fold across 79% of the genome and led to the identification of a large catalogue of high-confidence single nucleotide polymorphisms (SNPs), some of which not only confirmed his hair colour but also showed that he was of the A+ blood type and most likely had brown eyes, dry earwax, as well as a metabolism and body mass index adapted to cold climate. Admixture [23] and principal component analysis [24] of the SNP information indicated no affinity with modern-day Europeans, thus ruling out possible contamination, a problem that had plagued ancient human DNA studies for decades [25], as well as attempts at sequencing the Neandertal genome a few years earlier [26,27]. Analyses also revealed a much closer genetic affinity with contemporary Chukchis and Koryak populations of northeast Siberia, than with present-day Greenlandic Inuit. Divergence times with the Chukchi population closely matched the radiocarbon date for the ancient individual, suggesting that the Saqqaq ancestors entered Greenland soon after they separated from their Old World relatives and were later replaced by the ancestors of modern-day Inuits. This study demonstrated the immense potential of palaeogenomics towards reconstructing the population history of humans in much greater detail than what can be achieved from patterns of modern genomic variation alone.

A recent re-analysis of the Saqqaq sequences also revealed epigenomic signatures indicative of gene expression. Sequence depth variation showed a strong approximately 200 bp periodicity, which is characteristic of the length of one nucleosome and spacer block [28]. Within a shorter range, a 10 bp periodicity corresponding to the size of a DNA helix turn was also detected, reflecting preferential cleavage of the DNA backbone that faces away from nucleosome protection. Importantly, patterns of sequence depth variation at known nucleosome arrays showed strong correlations with nucleosome occupancy. It therefore seemed that in addition to the DNA sequence, the compaction state of the chromatin could survive in fossils, and that variation in read depth, corrected for base compositional bias, could be used as a footprint of nucleosome protection to reconstruct genome-wide nucleosome maps. Regional methylation levels could also be tracked in the Saqqaq genome sequence owing to the fact that it had been obtained by amplifying DNA libraries using a polymerase that amplifies cytosines deaminated due to post-mortem damage only when methylated [29–31]. Focusing on read starts, where deamination rates are highest, estimated methylation levels were found to recapitulate known genomic patterns at different classes of CpG promoters, splice sites and CTCF transcriptional repressor sites. Strikingly, the Saqqaq methylome also appeared closer to that of modern hair than to other somatic tissues. As nucleosome occupancy and cytosine methylation influence gene expression, those could be used to predict gene expression levels in the Saqqaq hair cells. As expected, key structural components of hair, such as keratins and trichohyalin, were predicted as highly expressed, demonstrating that ancient gene expression levels can be gathered directly from ancient sequence data, even in the absence of RNA. This approach can therefore complement functional SNP genotyping [32] and proteomics [33] to gather functional information from ancient individuals and investigate the dynamics and evolutionary significance of epigenomic changes.

## 4. Ancient anatomically modern humans

Recent mixed ancestries among modern human groups can limit our ability to infer their true past population history. Following the sequencing of the Saqqaq genome [22], a variety of ancient human genomic studies have shed light on major events in the human population history [34–41]. Of key importance, post-mortem DNA damage patterns and heterozygosity levels observed in the mitochondrial genome [42], or on the X-chromosome of male individuals, have provided robust approaches to rule out modern contamination.

Using 600 mg of hair collected in the 1920s from an Aboriginal Australian male, Rasmussen *et al*. [34] were able to reconstruct his genome at 6.4-fold coverage and found no evidence for recent European admixture or contamination. Genomic affinity was revealed with present-day Aboriginal Australians, as well as Bougainville and New Guinea Highland Papuans. More importantly, being the first Aboriginal Australian genome sequenced, this dataset allowed for the testing of two alternate models of modern human dispersal into eastern Asia. A statistics based on quartet genome alignments (*D_4P_*) showed an excess of genomic sites in support of a population tree where Aboriginal Australians and Africans cluster together, separate from Europeans and Asians. Population split times suggested that Aboriginal Australians separated from the ancestral Eurasian population around 62–75 kyr ago, which in turn radiated into the European and Asian branches some 25–38 kyr ago. These results favour the hypothesis of the ‘multiple dispersal’ model [43], which was also supported by the excess of shared derived alleles observed between Asians and Aboriginal Australians, reflecting population migration from the mainland. By contrast, the Australian Aboriginal genome lent little support to the alternative ‘single dispersal’ model proposing that humans expanded out of Africa into Eurasia 50 kyr ago [44] through a series of founder events, which ultimately gave rise to the colonization of Australia and the diversification of Aboriginal Australian populations.

Ancient human genomic data has also shed light on another fiercely debated topic in anthropology, namely the peopling of the Americas. Genomic signatures of an Upper Palaeolithic (approx. 24 kyr ago) male juvenile excavated at the Mal'ta site, south central Siberia, Russia revealed no strong connection with present-day eastern Asians [39]. Tree-based analyses of population splits and admixture events (Treemix [45]) instead placed the Mal'ta specimen basal to western Eurasians and identified gene flow from Mal'ta to Native Americans. Shotgun sequencing of another individual from the same region and dating to post-last glacial maximum (LGM; 17 kyr ago) showed a strong affinity with the Mal'ta specimen, suggesting that the population's gene pool was rather stable during the LGM, and consequently, that the populations from the region changed within the last 17 kyr. The western Eurasian component of the Mal'ta specimen suggests that Upper Palaeolithic populations ancestral to present-day western Eurasians had a distribution range that extended further northeast. This is consistent with the discovery of a number of anthropomorphic Venus figurines at the Mal'ta site, reminiscent of Upper Palaeolithic sites in western Eurasia. Interestingly, no particular genetic affinity was detected between present-day western Eurasians and a 40 kyr old individual excavated at the Tianyuan cave in north east China [38], confirming that the results were not affected by recent events in the population history of modern eastern Asians. The contribution of the Mal'ta lineage to the Native American gene pool gives further support to the hypothesis of a Siberian origin for present-day Native Americans, and the gene flow between Mal'ta and the ancestors of 52 Native American populations from Greenland to southern Chile was estimated to be responsible for 14–38% of the current Native American ancestry. This gene flow occurred before 12.6 kyr, which is the age of a child excavated at the Anzick site, Montana, USA, whose genome also revealed a ‘Mal'ta-like’ component [40]. The Anzick child belonged to the Clovis culture, the oldest archaeological complex in North America, and was part of a meta-population directly ancestral to all contemporary Native Americans outside of Canada and the Arctic. Overall, it appears that the ancestors of present-day Native Americans were the descendants of at least two population backgrounds, one related to the Mal'ta individual, showing a western Eurasian affinity, and another related to present-day eastern Asians, as suggested by the strong eastern Asian genetic component found among Native Americans [46]. This new model for the origin of Native Americans potentially solves the mystery surrounding the presence of non-east Asian morphological features in the skulls of the first Americans.

Ancient genomics has also provided invaluable clues to understand the complex genetic make-up of Europeans. The genome of a 5.3 kyr old Copper Age ‘Tyrolean Iceman’ revealed genetic discontinuity with current inhabitants of the Alps, with the Iceman showing a greater genetic affinity with southern European populations, and in particular with Sardinians [35]. This finding suggests that the current Sardinian population represents a remnant of an ancient and previously more widespread component of the European gene pool. Similarly, genome-wide data indicated that an approximately 5 kyr old early farmer from Sweden was more closely related to southern Europeans than to present-day northern Europeans and three contemporary hunter–gatherers of Sweden [36]. Those three hunter–gatherers as well as two 7 kyr old hunter–gatherers from the Spanish cave called La Braña [37,41] fell outside the present-day European genomic diversity but exhibited a closer genetic affinity with present-day populations of northern Europeans. One La Braña individual [41] was found to share ancestry with the Siberian Mal'ta individual, thus providing further evidence for the genetic and cultural links between the West Eurasian Mesolithic and the Siberian Upper Palaeolithic. All together, these results suggest a shared genomic background of hunter–gatherers across North Eurasia, as well as a migration-driven transition associated with the advent of the agricultural lifestyle in Europe.

Genomic data from ancient humans in Europe revealed information about their likely phenotypes and health status. For instance, the Tyrolean Iceman probably had brown eyes, belonged to the O+ blood group and was lactose intolerant. He was also homozygous for alleles associated with major risks for coronary heart disease and atherosclerosis, and infected with *Borrelia burgorferi*, the pathogen responsible for Lyme disease [35]. La Braña hunter–gatherers probably had difficulties digesting milk and starch, were dark haired and dark skinned and had non-brown eyes. The derived variants of immunity genes found in the La Braña genome suggested that hunter–gatherers were adapted to resist multiple types of infection that were commonly believed to have emerged much later with the advent of the agricultural lifestyle [41].

## 5. The genomics of archaic hominins

The Neandertal genome project represents a milestone in ancient genomics, as it led to major technical improvements, both for generating and analysing aDNA data [47]. Most of the final sequences used for the first genome draft assembly required no more than 400 mg of bone material sampled from three female specimens excavated at the Vindija cave, Croatia and dated to 38–44 kyr ago [47]. More recently, Prüfer *et al*. [48] generated a high-quality genome from a female Altai Neandertal from the Denisova cave, Russia, and a low-coverage draft genome from an approximately 60–70 kyr old Neandertal infant. The high-quality genome could be obtained owing to a combination of exceptionally high fraction of reads aligning to the human genome and minimal contamination levels. This vast genomic dataset makes the Neandertals the best-characterized extinct species today. The high-quality Altai genome revealed high inbreeding coefficients compatible with half-sibling mating, and temporal variations in the Neandertal population size, which was estimated to have been about a tenth of that of present-day humans, despite a broad Eurasian geographical range extending from the Iberian Peninsula to the Altai mountains.

Earlier genetic screening of a finger bone excavated at the Denisova cave had revealed the presence of an archaic hominin belonging to a mitochondrial lineage very distinct from modern humans and Neandertals [49]. Enzymatic treatment prior to DNA library preparation eliminated the vast majority of nucleotide misincorporations resulting from post-mortem damage, and further, employing paired-end sequencing and collapsing mate reads that showed sufficient overlap delivered a first draft of the nuclear genome with limited error rates [50]. The nuclear sequence data supported a different population scenario than the mitochondrial data. The archaic hominin appeared indeed to belong to a group distinct from both modern humans and Neandertals, but more closely related to Neandertals than to modern humans. This group was named Denisovans after the cave where it was first discovered. A molar tooth excavated at the Denisova cave contained enough endogenous DNA to reconstruct another full mitochondrial sequence using target enrichment. The latter appeared closely related to the sequence from the finger bone. The development of a new DNA library preparation method targeting single-stranded molecules enabled the reconstruction of an ancient genome showing a quality comparable to that of modern genomes sequenced at similar depth [51].

The temporal limits of archaic human genomics were recently pushed back with the sequencing of the complete mitochondrial genome of a 400 kyr old hominin from the Sima de los Huesos cave in northern Spain [52]. The sequenced individual is morphologically characterized as *Homo heidelbergensis*, a lineage commonly considered as pre-Neandertal. Yet, the mitochondrial genome appeared closer to Denisovans than Neandertals. Further genetic information, at the nuclear level, is required before this mitochondrial affinity can be confirmed or this result can alternatively be demonstrated as a consequence of a complex population history involving incomplete lineage sorting and/or gene flow [53].

The Neandertal and Denisovan genomes have revealed important information regarding admixture among archaic hominins and anatomically modern humans. *D*-statistics [54] indicate that Neandertals shared an excess of derived alleles with non-African modern populations [47–48,50–51]. This suggests that anatomically modern humans and Neandertals admixed in Eurasia. According to the latest estimates [48], this gene flow introduced 1.5–2.1% of Neandertal ancestry (most closely related to the individual from the Caucasus than the Altai) into the genome of non-African individuals. However, it is still debated whether the genomic patterns observed result from admixture between anatomically modern humans and Neandertals [55,56] or reflect ancestral population structure in Africa [57,58]. In contrast to Neandertals, Denisovans showed no evidence of gene flow into most present-day Eurasian populations, but did contribute to the gene pool of modern Melanesians [51,59] and, to a lower extent, of mainland Asian populations [48,60]. The admixture signature in present-day Papuans is greater on the autosomes than on the X-chromosome, possibly indicating the presence of hybrid incompatibility alleles on the X-chromosome, or that the gene flow preferentially involved Denisovan males and human females. Archaic hominin populations also appear to have mixed with each other. The level of Neandertal gene-flow into Denisovans is currently estimated at more than 0.5% [48] and an additional gene-flow into Denisovans originating from an unidentified hominin population (representing an outgroup to modern humans, Denisovans and Neandertals) has been proposed.

The archaic hominin genomes have importantly helped narrow down the genetic changes that make us humans [47–48,50–51]. Our understanding of how those relate to phenotype is, however, still in its infancy. In Denisovans, one difference was found in *EVC2*, a gene whose mutated alleles cause wider dental pulp cavities and fusion of tooth roots, both of which are common in the teeth of archaic hominins [51]. In Neandertals, some genetic variants in the *RUNX2* gene have been linked to cleidocranial dysplasia, which is associated with bell-shaped rib cages and changes in dental morphology, all of which represent major phenotypic differences between Neandertal and modern humans [47]. Functional assays also showed that the microRNA mir-1304 might be one factor involved in the difference in tooth morphology between modern humans and Neandertals [61]. The availability of the genome sequence allows anyone interested in a particular locus to investigate the variants present in archaic hominins, and potentially discover advantageous alleles that some modern human populations acquired from archaic hominins. Many such examples are now described and concern genes that are almost exclusively involved in the innate immune system (*STAT2* [62]; *OAS1* [63]; *HLA* [64]).

## 6. Towards Middle Pleistocene genomes and proteomes

With high-quality genomes from the Holocene and the Late Pleistocene in hand, the question soon became how far back in time palaeogenomics could be pushed. In 2012, deep sequencing of DNA extracts from a 110–130 kyr old bone delivered genome-wide information from a polar bear [65]. This suggested that, at least in cold environmental conditions, such as those found in the Arctic Ocean Svalbard archipelago where the polar bear material was discovered, palaeogenomics could break the Middle Pleistocene time barrier (125–781 kyr ago). At that time, the genetic evidence that DNA could survive over several hundreds of thousand years was rather scarce and limited to the pyrosequencing of no more than 16 bp of mitochondrial bases from approximately 400 kyr old cave bear specimens [66], PCR amplicon sequencing of minibarcodes from 450–700 kyr old ice cores [67] and 400–600 kyr old sediment cores [68]. Yet, successfully sequencing Middle Pleistocene genomes would provide much needed perspectives across a broad range of evolutionary biology questions. Not less than five archaic hominins were living during the Middle Pleistocene [69], including the most recent common ancestor of anatomically modern humans, Neandertals and Denisovans. The Middle Pleistocene also experienced numerous radiations and extinctions of fascinating megafauna lineages [70,71], as well as major climatic changes, involving the succession of many glacial and interglacial episodes, in contrast to only one for the Late Pleistocene. Clearly, pushing the limits of palaeogenomics to the Middle Pleistocene would represent a major step forward.

The empirical demonstration that such a step is possible came from a fragment of horse metapodial bone excavated in 2003 at Thistle Creek (TC), Yukon, Canada [72]. The specimen was found within a stratigraphic layer associated with the Gold Run tephra and dated to 735 ± 88 kyr BP, in agreement with palaeobotanical and micromammal fossil analyses, which also indicated an Early–Middle Pleistocene age [73–75]. The line of evidence suggesting that biomolecules, including DNA, could survive for such a long time included: (i) the detection of amino acids within the bone matrix by time of flight secondary ion mass spectrometry, (ii) the identification of the three most abundant amino acids in the primary sequence of collagen (glycine, proline and alanine) in the bone matrix, (iii) the direct sequencing of a variety of peptides representing 72 proteins from the bone matrix and the circulating blood, (iv) the presence of significantly greater levels of protein degradation by glutamine deamidation in the TC horse than in a younger Late Pleistocene Siberian mammoth, and (v) the estimation of considerably higher levels of DNA damage in the TC horse than in younger Late Pleistocene horses also preserved in the Arctic permafrost. Additionally, phylogenetic inference based on complete mitochondrial genomes revealed that the TC horse fell outside the range of genetic variation of modern and Late Pleistocene horses. This was confirmed using the full set of protein-coding nuclear genes and a total of eight other genomes sequenced for comparison, including a 43 kyr old horse [72]. In addition, the retrieval of genomic information from Middle Pleistocene specimens is compatible with the long-term survival of DNA predicted by the empirical model of DNA degradation through time proposed in [76].

The TC horse genome was sequenced at approximately 1.1-fold coverage using a combination of second-generation (Illumina) and third-generation (Helicos) sequencing. The latter, based on true single DNA molecule sequencing (tSMS), appeared to be advantageous when targeting short and damaged molecules for several reasons. First, this technology is PCR-free and, thus, devoid of PCR-related bias. Second, it does not require extensive enzymatic manipulation or repeated DNA purification steps, thereby maximizing DNA recovery and reducing the risks of enzyme incompatibility with chemically modified ancient templates. Third, this technology operates with single DNA strands and from any available 3′-hydroxyl group available. Consequently, with higher densities of single-strand breaks compared with modern DNA, aDNA templates present a higher chance of being sequenced on this platform. Methodological improvements of the sequencing protocol [77,78] and the development of dedicated bioinformatics strategies to improve the sensitivity and accuracy of read alignment against the horse reference genome [79] were necessary to optimize the analysis of aDNA molecules using tSMS. Altogether, this resulted in the identification of 4.21% of Helicos reads as endogenous horse DNA versus only 0.47% for Illumina reads.

The TC horse genome sequence was used first to date the time of the most recent common ancestor of horses, donkeys, zebras and asses at approximately 4.0–4.5 million years (Myr), which corresponds to twice the age of the first widely accepted *Equus* fossil from the palaeontological record. This new calibration point provided a genome-wide mutation rate that was used for scaling the palaeodemographic profile reconstructed from high-quality modern diploid genomes following pairwise sequentially Markovian coalescent inference. The profile revealed three major periods of demographic expansions and contractions for horses within the last 2 Myr, the last of which was consistent with ecological niche modelling and palaeoenvironmental data showing grassland expansion prior to the LGM followed by a massive post-LGM range contraction [80]. Interestingly, Bayesian skyline reconstructions based on the ancient mitochondrial genomes sequenced in this study, as well as tip-calibration, showed similar demographic changes, providing an independent validation of the novel calibration point proposed for *Equus*.

More fundamentally, the sequence data provided a unique snapshot of aDNA molecules from the Middle Pleistocene, revealing for the first time the presence of 3′ overhangs [77] and fragmentation levels compatible with the survival of ultra-short fragments (25 mers) over 1 Myr. As the latter provide sufficient information for mapping, environmental conditions close to those in place at TC should therefore enable the characterization of 1 Myr old genome [81]. Outside the Arctic, temperate caves that represent an environment with virtually no variation in temperature are also likely to offer preservation conditions compatible with the reconstruction of Middle Pleistocene genomes, as shown recently by the analysis of bone material from Sima de los Huesos at Atapuerca [52,82]. In these studies, the experimental procedure, which combined a newly developed extraction method tailored to the retrieval of ultra-short DNA fragments, single-strand Illumina DNA libraries [51,83] and target-enrichment capture, recovered enough high-quality DNA reads to reconstruct a near complete mitochondrial genome of a Middle Pleistocene cave bear and *H. heidelbergensis* [52].

## 7. Non-mammalian palaeogenomics

Despite major advances in genomic technologies, assembling complete plant genomes is a major challenge even for modern samples owing to their large, highly repetitive and heterozygous genomes, confounded by varying ploidy-levels, even within genera. Thus small-scale plant aDNA studies have been undertaken on maize [84], barley [85], cotton [86], wheat [87] and bottle-gourd [88], revealing patterns of crop adaptation and migration (reviewed in [89]). Larger and more in-depth studies using ancient plant genomes are expected in the coming years given the economic importance of major crops and the possibility of reintroducing alleles involved at various stages of the domestication process. Herbarium collections hold great potential as resources for future investigations of historical genomics as the specimens are generally well preserved and often meticulously annotated. High endogenous DNA content has allowed the genomic characterization of ‘ancient’ genomes of the plant pathogen *Phytophthora infestans*, the oomycete responsible for the Irish potato famine [90–92]. Herbarium collections also hold potential for population genomics. Future studies on ancient plant pathogens should reveal more details about their coevolution with food crops and the history of their human-mediated migrations, potentially leading to insights for crop breeding and management. Furthermore, the study of aDNA from non-domesticates such as forest trees will probably shed light on changes in biodiversity during past climatic events [93].

It has also recently been shown that RNA is preserved in some ancient seeds, even better than aDNA in maize kernels [94], presenting an opportunity to directly test evolutionary changes in gene expression at a key developmental stage [94]. Comparative genomic approaches and selection scans will also probably narrow a series of candidate loci that were adaptive in a range of environmental conditions. It should also be stressed that new aDNA reservoirs, such as egg shells [95] and dental calculus [96,97], are constantly being discovered, and should greatly benefit from deep-sequencing approaches.

## 8. Ancient genomes: a user's manual

### (a) DNA damage patterns as authenticity indicators

With the introduction of genomics, new authentication criteria for DNA have emerged, with one of the most essential being the detection of typical signatures of post-mortem damage. Using blunt-end ligation of double-stranded templates, cytosine deamination at 5′-overhangs results in greater C → T misincorporation rates towards sequence starts [98]. At read ends, this signature is converted into a complementary increase in G → A misincorporation rates. This typical post-mortem damage signature is modified depending on the molecular tools used during library building and amplification. AT-overhang ligation, for instance, was shown to introduce significant biases in the sequence composition of library inserts, which discriminates against templates starting with thymine residues [99] and, consequently, deaminated cytosines (molecular analogues of thymines). This not only reduces the molecular complexity of DNA libraries, but also transforms the expected nucleotide misincorporation pattern, which peaks at the second position from sequencing termini. Likewise, DNA polymerases of the *Pfu* family, such as *Phusion*, cannot bypass uracil residues [22]. As a result, no increase in C → T misincorporation rates are detected at sequence starts [31], except at methylated CpGs [29].

**Figure 1.**
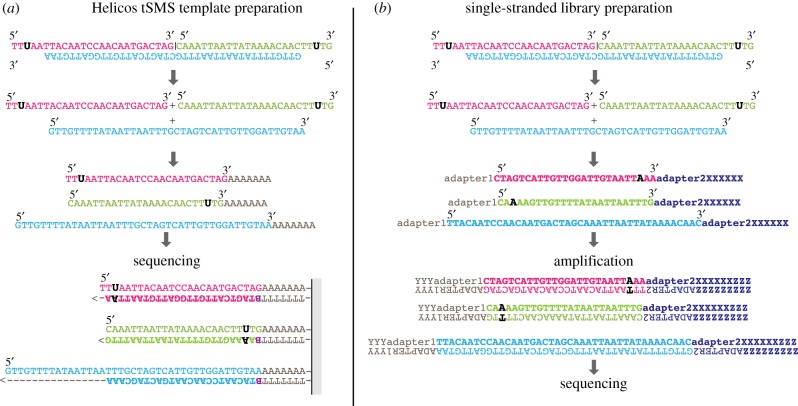
Accessing single-strand information from aDNA templates. Comparison of template preparation for (*a*) Helicos tSMS [77] and (*b*) single-stranded library building [51], in the case of a double-stranded DNA molecule showing a single-strand break ‘|’ and two cytosines deaminated to uracils (U). Both methods lead to three sequence reads, each represented by a different colour (pink, blue and green), thus providing strand information. Conversely, double-stranded molecules with a single-strand break built into double-stranded libraries lead to the sequencing of one read. Cytosine deamination in overhangs leads to an artefactual increase in G → A transitions in Helicos tSMS sequence reads and C → T transitions in sequence reads obtained from single-stranded libraries. (*a*) ‘B’, dCTP, dGTP or dATP virtual terminator. (*b*) ‘X’, ‘Y’ and ‘Z’ adapter/primer sequence.

Procedures based on single-stranded templates also show a different nucleotide misincorporation profile. With Helicos tSMS, a sequence reverse complementary to the original template strand is generated by extension from the blocking site (figure 1*a*). Cytosine deamination at 3′-overhangs of the template strand will, therefore, increase G → A instead of C → T misincorporation rates [77,78]. With single-strand Illumina DNA libraries [51,83], whenever inserts are paired-end sequenced or sequenced over their full length, the expected misincorporation pattern corresponds to an increase of C → T rates at both sequence starts and sequence ends (figure 1*b*).

One type of DNA damage pattern, where the genomic position located upstream of sequence starts is enriched in purines [98], appears to be common to all library building protocols. This probably reflects a mostly depurination-driven post-mortem DNA fragmentation process. Preferential loss of adenine over guanine residues has been observed for aDNA extracts younger than a century [100], but guanine residues are preferentially lost for much older material, suggesting two temporally independent depurination dynamics at adenine and guanine residues. The resonance structure present within guanine residues and reducing the activation energy required to break the bond with the deoxyribose might influence these dynamics [101].

Restricting analyses to the population of sequences exhibiting typical damage patterns has been shown to enable genuine data recovery, even in the presence of significant levels of contamination [39,52,102]. Nucleotide misincorporation and DNA fragmentation patterns can be detected using ad hoc programs such as the mapDamage software [103,104], and post-mortem degradation parameters can be quantified from read alignments against reference genomes [103,104]. In light of the versatility of the signatures described above, we recommend that the same molecular methods should be used when comparing DNA damage parameters across a range of samples.

### (b) Limiting the impact of post-mortem DNA damage

Fitting a DNA damage model to the data can also be used to limit the impact of C → T and G → A misincorporations in downstream analyses. In particular, the confidence placed on any nucleotidic base along a sequence can be downscaled post-mapping according to the probability of the base being affected by post-mortem damage [104]. This approach was shown to reduce the false positive rate of SNP calls on the Saqqaq data [104]. Ideally, the damage model should be applied during the mapping step itself, in order to improve read alignment accuracy and sensitivity, as currently implemented in the programs MIA [105], ANFO [47] and sesam [22], but usage of these softwares has been limited mostly owing to long-running times (MIA) and the inability to handle indels (sesam).

For now, the most common strategy for limiting the impact of misincorporations in downstream analyses has consisted in a first authentication of the data based on a small subset of sequences followed by a second production phase using nucleotide misincorporation-free libraries [47,106–108]. This is done by treating the aDNA extract with a cocktail of two enzymes, where the uracil-DNA glycosylase targets deaminated cytosines and generates abasic sites, which represent the targets for the EndoVIII endonuclease [29]. As a result, shorter DNA templates cleaved at damaged sites are ligated to adapters and incorporated into libraries. This strategy, in addition to high depth-of-coverage and paired-end sequencing where almost every single base position of the insert is read twice, has been essential for generating the high-quality Denisovan genome sequence [51].

### (c) Increasing the relative amount of target DNA I: ancient DNA extraction

While standard silica-based extraction procedures [108] are biased towards molecules longer than approximately 40 bp, a recent extraction method has been developed to target the ultra-short fraction of DNA extracts [82]. Assuming that DNA fragmentation follows a one-order kinetics, the amount of aDNA available decreases exponentially with fragment size [76,109]. By targeting short DNA fragments, the new extraction method should thus drastically increase the amount of aDNA material in extracts, thereby increasing the molecular complexity of DNA libraries. This method is an important advance towards the sequencing of significantly older DNA templates, as well as DNA from environments offering poor preservation conditions. Methods have been devised for the preferential extraction of DNA from molecular preservation niches that can be found within fossils. Such niches have been proposed to correspond to crystal aggregates present in the most interior parts of bones where endogenous DNA is protected from hydrolysis and microbial invasion [110]. One promising method has shown great success with permafrost preserved bone material, such as mammoth [111] and horses [77,78,112]. This method involves a first partial digestion of the bone powder, before undigested bone pellets are recovered and digested a second time in a fresh buffer. Pairwise tests have shown higher endogenous contents in the extract prepared from the second digest, as well as lower levels of cytosine deamination [77,78,112] and fragmentation [78]. If confirmed by additional tests on a range of ancient samples, targeting such molecular preservation niches could significantly reduce the costs related to ancient genome sequencing.

### (d) Increasing the relative amount of target DNA II: ancient DNA library construction and amplification

Apart from library construction methods [99], the type of DNA polymerase used also significantly impacts the complexity of amplified DNA libraries [113]. Standard polymerases for aDNA research, such as *Taq* Gold, significantly skew the size distribution and base composition of the pool of molecules amplified towards short and GC-rich templates, which limits the ability to sequence the entire molecular diversity originally present in the DNA library. DNA polymerases can also be blocked during library amplification owing to the presence of atypical bases in aDNA templates [114], which can in turn provide an advantage to non-modified modern contaminants. The exact amount of such blocking DNA lesions is still largely unknown but would represent 10–40% of library templates according to a recent estimate using a limited number of fossil specimens from permafrost and temperate caves [114]. Devising methods for repairing such templates and/or capturing preferentially damaged templates [53] could further improve accessibility to aDNA molecules. DNA polymerases capable of bypassing damage lesions in DNA molecules have been engineered and shown to significantly increase amplification success from Pleistocene specimens, but their efficiency has not been investigated yet with next generation sequencing approaches [115].

### (e) Increasing the relative amount of target DNA III: ancient DNA enrichment

**Figure 2.**
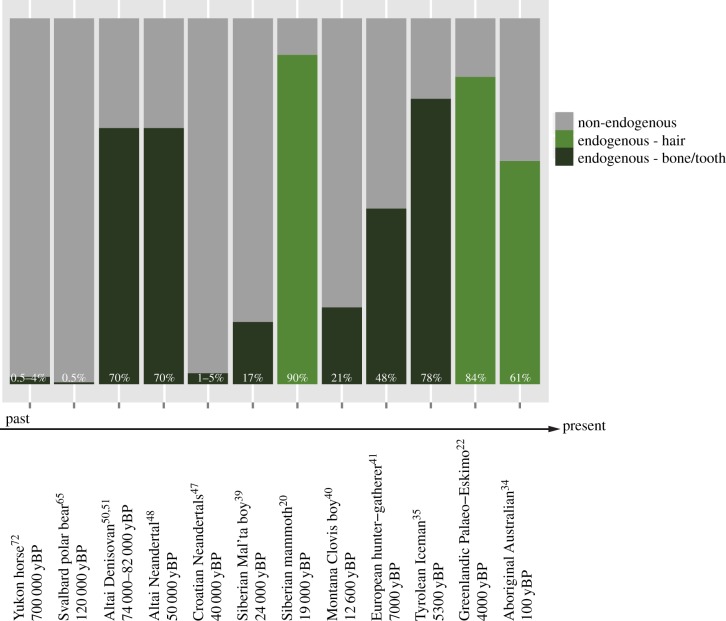
Endogenous content of ancient genomic extracts. Datasets are ordered from the most ancient sample to the most recent. ‘yBP’, years before present.

The fact that most aDNA extracts show a minority of endogenous templates (figure 2) has led to the development of enrichment approaches aimed at reducing sequencing costs and improving the sequence quality of the targeted loci. Primer extension capture was the first of such methods and succeeded in recovering full mitochondrial sequence information from five Neandertal specimens [116] and from an approximately 30 kyr old modern human [117]. It was later superseded by other in-solution enrichment methods relying on biotinylated baits, either designed from known sequences and manufactured commercially [118], or prepared from modern DNA extracts [119]. These baits are subsequently used to target complementary library inserts. Both methods have shown great success in various aDNA contexts, often delivering complete mitochondrial genome sequences with high depth of coverage [42,72,117,119–123] and even pre-selected regions from the *Mycobacterium tuberculosis* genome [124]. The method has also been found to be relatively robust to the evolutionary distance separating probes and targets, yielding to significant enrichment despite 10–13% of sequence divergence [125]. Microarray-based hybridization capture has also performed well in enriching Neandertal DNA libraries [32] containing very low endogenous DNA content, and delivering the full bacterial genome of the causative agent of the mediaeval Black Death epidemic [106,126] as well as of historical leprosy strains [127]. One drawback of such approaches is that microarrays are designed from modern reference genomes. As a consequence, untargeted plasmids and/or loci potentially present in the historical strains and/or chromosomal rearrangements specific to the historical strain could remain undetected. This problem can be solved by using de novo genome assembly in cases where samples with exceptionally high pathogen DNA contents are available, such as for one of the three ancient leprosy samples that were genome sequenced [127].

Other types of enrichment approaches have been developed to target full human chromosomes [38], and even complete genomes [128,129], which performed well on poorly preserved DNA material. One such approach converts custom-designed microarray probes into an immortalized and amplifiable biotinylated library of baits that can be used for pulling-down orthologue inserts from aDNA libraries. This strategy enabled Fu *et al*. [38] to reconstruct all non-repetitive sequences of chromosome 21 in a 40 kyr old anatomically modern human from Tianyuan cave, China, from a set of immortalized baits recovered from nine microarrays and corresponding to 8.7 million probes across 30 Mb of the chromosome. A second approach achieves full genome enrichment in solution with no prior need for microarray purchase, therefore cutting down on prohibitive microarray costs [128,129]. Here, modern DNA from a given organism is first built into a DNA library downstream of RNA polymerase T7-promoters so that genome-wide biotinylated RNA baits can be transcribed *in vitro*. Baits are then used to pull down orthologue inserts from regular aDNA libraries, followed by several washing steps to rid the library of exogenous DNA library inserts. This method has shown twofold to 13-fold enrichment in human DNA content, which provided enough SNP information for population assignment with minimal sequencing effort. Up to 19-fold enrichment was obtained on mammoth aDNA extracts, thus demonstrating that this approach can be applied on extinct species, even in absence of reference genomes [129]. Sequencing of genome-wide captured libraries is cost-effective, and thereby well suited for the analysis of large numbers of samples, which promises to move forward the field towards population genomics. aDNA whole genome enrichment and the development of capture methods targeting damaged DNA molecules will probably facilitate the characterization of Middle Pleistocene genomes in the near future.

## 9. What next?

Looking back 5 years, no one could have predicted the current state of ancient genomics. New sequencing technologies requiring no heavy infrastructure are being developed with the promise of delivering gigabases of sequence information at small cost. We can anticipate that ancient genomics will move on to the scale of population studies, with probable research areas in the reconstruction of human dispersal routes and demographic processes, such as the ones associated with the Neolithic transition in Europe. In addition, we expect that palaeogenomics will soon help better understand the origins, evolution and pathogenicity of the bacterial and viral agents responsible for major historical pandemics in human history. Ancient genomics will also probably illuminate the domestication process by revealing the genes that have been artificially selected to transform wild animal and plant species into the variety of domesticated forms that we know today. Additionally, high-quality ancient genomes of megafaunal species together with genome-wide SNP surveys will document past demographic trajectories at unprecedented levels [130]. This type of approach will complete our current understanding of how species responded to major climatic changes in the past [80], a key to conservation genomics in the face of current global warming. Moreover, the accumulation of aDNA data will probably provide additional information on post-mortem DNA base modifications, which will be essential for understanding and correcting ancient genomic datasets, and also for reconstructing ancient methylation marks. Finally, we predict advances in the recovery of functional information from ancient specimens through proteomics [131], which has already delivered partial proteomes from extinct mammalian species [33,72], and through ancient epigenetic marks and nucleosome maps [28]. There is little doubt that, together, these prospects will catalyse yet another revolution in aDNA research.
